# Effectiveness of Meta-Cognitive and Cognitive-Behavioral Therapy in Patients with Major Depressive Disorder

**Published:** 2013

**Authors:** Ahmad Ashouri, Mohammad Kazem Atef Vahid, Banafsheh Gharaee, Maryam Rasoulian

**Affiliations:** 1 Clinical Psychologist, Assistant Professor, Tehran Institute of Psychiatry and Mental Health Research Center, Iran University of Medical Sciences, Tehran, Iran.; 2 Psychiatrist, Associate Professor, Tehran Institute of Psychiatry and Mental Health Research Center, Iran University of Medical Sciences, Tehran, Iran.

**Keywords:** Cognitive Behavior Therapy, Major Depression Disorder, Metacognitive Model, Metacognitive Therapy

## Abstract

**Objective:** The present study aimed to compare the effectiveness of metacognitive therapy (MCT) and cognitive-behavior therapy (CBT) in treating Iranian patients with major depressive disorder (MDD).

**Methods:** Thirty three outpatients meeting DSM-IV-TR criteria for MDD without any other axis I and II disorders were randomly assigned to one of three treatment conditions, i.e. MCT, CBT and pharmacotherapy. The Beck Depression Inventory-II-Second Edition (BDI-II), Beck Anxiety Inventory (BAI), Ruminative Response Scale (RRS) and Dysfunctional Attitude Scale (DAS) were administered for pre-treatment, post-treatment and follow-up. Data were analyzed by repeated measures analysis of variance (ANOVA).

**Results: **Based on repeated measures ANOVA, all the participants demonstrated improvement in depression, anxiety, dysfunctional attitude and ruminative response. Based on percentage results, all the patients in MCT and CBT groups showed significant improvement at post-treatment phase.

**Conclusions:** MCT and CBT were more effective than pharmacotherapy alone In treatment of MDD.

**Declaration of interest:** None.

## Introduction

Depression is one of the most prevalent psychiatric disorders which imposes high economic, emotional and social burden on patients, families and society (1). Approximately 121 million people suffer from depression worldwide (2). Currently, depression ranks fourth among the ten leading causes of global disorders costs , and it is predicted that it will be the second leading cause of financial burden globally by 2020 (3). Studies have also showed that prevalence of depression among Iranians is quite high (4-6). 

Concerning high prevalence and distasteful consequences of depression, effectiveness of different types of drugs and psychological interventions on depression has been investigated**. **During the past three decades, about 200 studies have compared the effectiveness of psychological interventions with controlled situations and other therapies (7). Results have demonstrated the effectiveness of psychological interventions in treatment of depression (7-9). 

One of the most common psychological interventions is cognitive-behavioral therapy (CBT) which its effectiveness has been confirmed in different studies (10). In some cases, CBT was considered as alternative treatment for depression (11, 12). The theoretical basis of CBT in depression originates from the behavioral and cognitive theories of depression. Beck’s theory (13) is the most important and widely recognized cognitive theory of depression. In this approach, the negative thoughts may cause depression in people. According to Beck, depression is resulted from individual’s negative views of ego, world and future which form a cognitive triangle. It is assumed that if negative schemas become active, they would produce cognitive biases with the tendency to process information negatively, thus leading to low and reduced mood (14).

In conclusion, it can be mentioned that Back’s approach gives priority to negative beliefs and attitudes in reducing mood. The cognitive approaches try to treat depressed patients through changing the cognitive content of their thoughts. Although studies have shown that cognitive behavior therapy is the most effective psychological treatment for major depression (11, 12); however, this approach did not address the therapeutic needs of all patients. The outcome studies using Beck’s Depression Inventory (BDI) have reported that only 40-58% of patients show improvement without any relapse at the end of the treatment (15, 16). 

Recently, new approaches including meta-cognitive theory (MCT), have been proposed which gives priority to mood in producing negative thoughts, beliefs, and attitudes (17). Self-regulatory executive function model, also known as S-REF, developed by Wells and Matthews (18, 19) was the first model that conceptualized the role of meta-cognition in provoking mental pathologies and disorders. In fact, psychological disorders are sustained when maladaptive coping strategies such as anxiety, rumination, threat monitoring, avoidance, and thought suppression, prevent the modification of dysfunctional self-beliefs, thereby increasing the availability of negative information towards ego (20).

MCT is one of the newest approaches in the field of clinical psychology. Its effectiveness in treatment of various psychiatric disorders has been confirmed through a number of well-controlled studies (21-23). MCT is a type of cognitive therapy using thought modification but is different from cognitive therapy in its conceptualization of specific disorders. The beliefs which are important in MCT including normal cognitions as negative automatic thoughts are not accounted in cognitive-behavioral therapies. However an individual’s beliefs about thinking determine meta-cognitive beliefs (24). 

The Meta-cognitive beliefs are said to be some beliefs that individual considers them about their experiences, thoughts and procedures (24, 25). MCT aims at replacing rumination process with negative automatic thoughts. MCT emphasizes on meta-cognitive knowledge and procedure differing from cognitive therapy in applying therapeutic techniques. MCT is recommended for mental disorders including generalized anxiety disorder (22), social anxiety disorder (26, 27), post traumatic stress disorder   (23, 28, 29) and obsessive compulsive disorder (30-35). A case study confirmed the effectiveness of MCT on depressed patients as well (36). 

No study has been done yet for comparing the effectiveness of this therapeutic approach with other approaches. The current study investigated the effectiveness of MCT versus CBT in treatment of major depressive disorder (MDD).

## Materials and Methods

This was an experimental study with three groups, i.e. two experimental and one control groups. The subjects were randomly assigned into the groups. 

Subjects of the first experimental group received meta-cognitive therapy in addition to their usual medication. The second group underwent cognitive-behavioral therapy (CBT) plus medication, and the control group received mere medication. 

Pretest and posttests were done on all the study subjects. Assessments and treatments were administered in outpatient setting by a PhD student of Clinical Psychology. 

The study design can be shown as the follows:

EG1           O_1_           X**         O_2_

EG2           O_3_           X*          O_4_

EG3           O_5_           X            O_c_

EG1, EG2 and EG3 represent two experimental and control groups, respectively.

 O1, O3, and O5 represent pre-tests of the three groups, and O2, O4, and O6 denotes post-tests of the groups. The X** shows MCT, X* indicates CBT, and X represents no treatment (control). 

The subjects were diagnosed by a psychiatrist and a clinical psychologist through psychiatric, as well as, structured clinical interviews. The mixed repeated measures analysis of variance (ANOVA) was applied for data analysis using SPSS for Windows 19.0 (SPSS Inc., Chicago, IL, USA) by a statistician unfamiliar with the study groups.

*Population and Sampling*

The population included patients with MDD. Goal-oriented and convenience sampling were used for selecting participants among patients who had been referred to university and private outpatient clinics in Tehran, Iran. Subjects of the study were comprised of 33 people who had been referred to the aforementioned centers. They had the following inclusive criteria:

-Having diagnosis criteria for MDD according to the results of structured clinical interview for DSM-IV, axis I, clinical version (SCID-I/CV) determined by psychiatrist and psychologist. 

-Receiving no psychological therapies during six months before participation in the study.

-Age between 18-50 years.

-Literacy level of at least third grade of guidance school.

-And signing the informed consent for participating in the study.

B-The exclusive criteria were as the follows:

-Having psychotic symptoms, drug abuse and other psychological disorders at Axis I according to the results of diagnostic interview and results of the SCID-I/CV determined by psychiatrist and psychologist as well as having serious suicidal thoughts as they have not good compliance.

-Having complete criteria of personality disorder at Axis II determined by psychiatrist and psychologist through diagnostic interview and results of the SCID-II test.

***Instrument***

*Structured Clinical Interview for DSM-IV Axis I disorders SCID-I*

Structured clinical interview for Statistical Manual of Mental Disorders, 4^th^ Edition (DSM-IV) Axis I disorders SCID**-**I (Structured Clinical Interview for DSM-IV), clinical version (SCID-I/CV) is a comprehensive and standardized instrument for assessment of major mental disorders in clinical and research settings (37). SCID**-**I is administered in a single session and takes about 45 to 90 minutes. Validity and reliability of this instrument have been confirmed in several studies (38). Zanarini et al. (39) has been reported inter-rater diagnostic reliability with Kappa higher than 0.7 in most cases. The Persian version of this questionnaire has been provided by Sharifi et al. (40). Validity of the instrument has been confirmed by clinical psychologists and its retest reliability was 0.95 for one week.

*Structured Clinical Interview for DSMIV Axis II disorders SCID-II*

Similar to SCID-I, SCID**-**II is a structured diagnostic interview for personality disorder to assess ten personality disorders at DSMIV Axis II, depressive and aggressive disorders in part of NOS (Not otherwise Specified) which were suggested by Forest, Gibbon, Williams, First et al. (41). This questionnaire has 119 questions, takes less than 20 minutes and requires literacy level of at least eighth grade. The interviewer conducted the interview on the basis of positive responses of the patient (41). 

An investigation has been conducted with 284 subjects from four psychiatric centers and two non-psychiatric centers by two interviewers at two different times in order to determine the test retest reliability in a two-week interval and during two different times. The Kappa coefficient was 0.24 for OCD, 0.74 for Histrionic personality disorder and 0.53 for all psychiatric patients. The inter-rater agreement was low (Kappa = 0.38) among non-psychiatric patients (41). 

The content validity of the Persian version has been confirmed by some psychological professors and its reliability was 0.87 through test-retest with a one-week interval (42).

*Beck* *depression inventory, second edition (BDI-II)*

The Beck depression inventory, second edition (43) is the revised Beck depression inventory (BDI) which was designed to assess the severity of depression in adolescents and adults (43). Compared to the first edition, the second edition of Beck inventory is more compatible with DSM-IV. In fact, it covers all depression items based on the cognitive theory. Cronbach's alpha was 0.86 and internal consistency coefficient was 0.92 among the U.S. people (43) and 0.91 and 0.94 among Iranian people, respectively (44).

*Beck Anxiety Inventory (BAI)*

Beck anxiety inventory (BAI) is a self-report inventory with 21 items designed to evaluate the severity of physical and cognitive symptoms of individuals during the last week. The score of each item ranges from 0 to 3 and the highest overall score is 63. The BAI has shown good test-retest reliability after 1 week following initial administration (α= 0.75) (45) and also good internal consistency (0.87) (46) and validity (45). A study (47) showed that in Iran, BAI had a good reliability (r = 0.72), a very good validity (r = 0.83) and an excellent internal consistency (α = 0.92) 

*Ruminative Responses Scale (RRS)*

Ruminative Responses Scale (RRS) is a self-report scale with 22 items designed by Nolen-Hoeksema and Morrow (48) to evaluate mental ruminations and tendency to ruminate in response to depressed mood. Questions of this scale are based on the concept of rumination and thoughts related to the depressed mood. The responses are scored based on a Likert scale ranging from 1 to 4. Using Cronbach's alpha, its validity coefficient ranged from 0.88 to 0.92 (49) and its test-retest was 0.67 during 12 months (50)**. **The Cronbach's alpha was reported to be 0.90 among Iranian subjects (51).

*Dysfunctional Attitude Scale (DAS)*

Dysfunctional Attitude Scale (DAS) is a commonly used self-report measurement of fundamental cognitive attitudes of Beck’s theory for depressive symptoms. The scale has 40 items in two parallel forms which are rated on a 7-point Likert scale ranging from 1 (Not True) to 7 (Very True). The DAS has demonstrated satisfactory reliability (α=0.85) and validity in previous studies. One study evaluated DAS in Iranian subjects and confirmed its factor structure and showed that the DAS test-retest reliability and internal consistency for total score were 0.90 and 0.75, respectively, and the correlation between DAS and BDI-II was 0.65 (52).

## Results

Subjects of the study included 33 patients at pretest (10 patients in MCT group, 10 patients in CBT group and 13 patients in the control group). 60.6% of the participants were female and 39.3% were male. Mean age of the patients was 32.48 years (± 7.71).

Table 1 illustrates the mean and standard deviation (SD) of the control and experimental groups on the depression, anxiety, dysfunctional thoughts and rumination scales in pretest, posttest and follow-up sessions. The results indicated that the mean and SD of the groups on all scales were close to each other at the pretest. Following the intervention, the mean scores of the experimental groups showed statistically significant changes compared to the control group. These changes were maintained at the follow-up session. 

**Table 1:** Mean and standard deviation of Beck Depression Inventory-II-Second Edition (BDI-II), Beck Anxiety Inventory (BAI), Dysfunctional Attitude Scale (DAS) and Ruminative Response Scale (RRS) in pre-test, post-test and follow-up

**Scale**	**Group**	**Pre-test**		**Post-test**		**Follow-up**			
		**Mean**	**SD**	**Mean**	**SD**	**Mean**	**SD**		
**BDI-II**	MCT	37.10	4.04	16.10	2.51	17.88	2.66		
	CBT	34.40	5.50	18.30	4.62	18.25	4.83		
	Control	34.30	6.54	25.55	3.55	28.14	2.41		
**BAI**	MCT	26.50	5.94		12.00	2.82	14.44	3.20	
	CBT	25.80	6.08	14.20	4.18	13.25	1.58		
	Control	25.92	07.86	19.44	3.08	20.28	3.72		
**DAS**	MCT	205.80	43.37	112.40	10.84	121.33	9.13		
	CBT	177.90	50.72	97.80	12.68	105.12	4.88		
	Control	208.30	36.72	140.55	3.35	150.00	12.31		
**RRS**	MCT	57.10	04.35	13.50	4.17	15.33	0.86		
	CBT	56.40	3.30	17.30	1.76	19.87	3.64		
	Control	57.92	2.28	2466	2.54	27.28	3.90		

MCT: Metacognitive therapy; CBT: Cognitive-behavior therapy

**Figure1.**
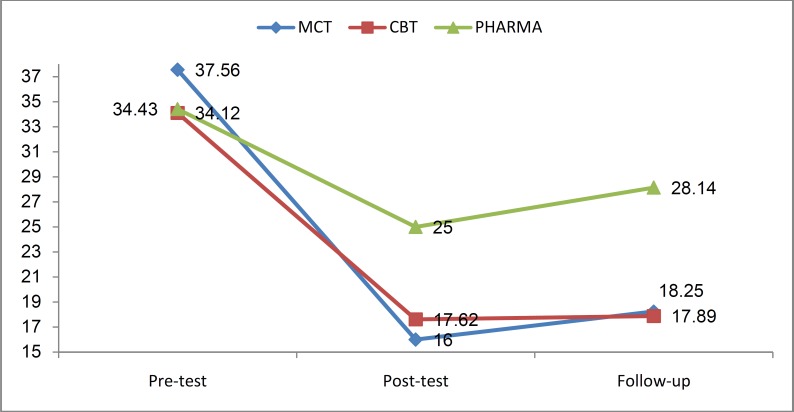
Comparison the adjusted mean of Beck Depression Inventory-II-Second Edition (BDI-II) in metacognitive therapy (MCT), cognitive-behavior therapy (CBT), and no psychotherapy groups during three phases of the study

The result of mixed repeated measures ANOVA demonstrated a significant interaction effect between phase and groups (F_(4,42) _= 39.37, p = 0.001, η^2^ = 0.48). According to the figure 1, as well as the results of the Post hoc tests for paired comparisons with Bonferroni correction, there were no statistically significant differences in depression scores between the groups at pre-test phase. In other words, this indicates the homogeneity of the groups in terms of depression scores. At post-test however, statistically significant differences were observed between the experimental (MCT and CBT) and control groups (p < 0.01). Similar results were noted at the follow-up stage. 

**Figure 2.**
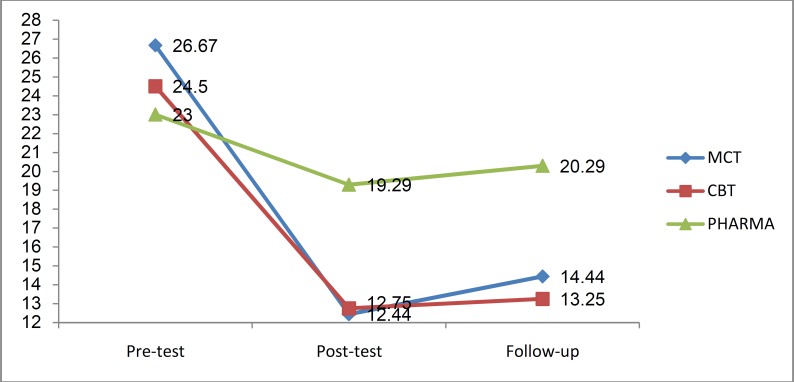
Comparison the adjusted mean Beck Anxiety Inventory (BAI) in metacognitive therapy (MCT), cognitive-behavior therapy (CBT), and no psychotherapy groups during three phases of the study

Results of mixed repeated measures ANOVA demonstrated a significant interaction effect between phase and groups (F _(4, 42)_ = 3.5, p = 0.05, η^2^ = 0.25). According to the figure 2, as well as results of the Post hoc tests for paired comparisons with Bonferroni correction there were no statistically significant differences in anxiety scores between the groups at pre-test phase. In other words, this indicates the homogeneity of the groups in terms of anxiety scores. At post-test however, significant differences were observed between the experimental (MCT and CBT) and control groups (p < 0.01). Similar results were noted at the follow-up stage.

**Figure 3:**
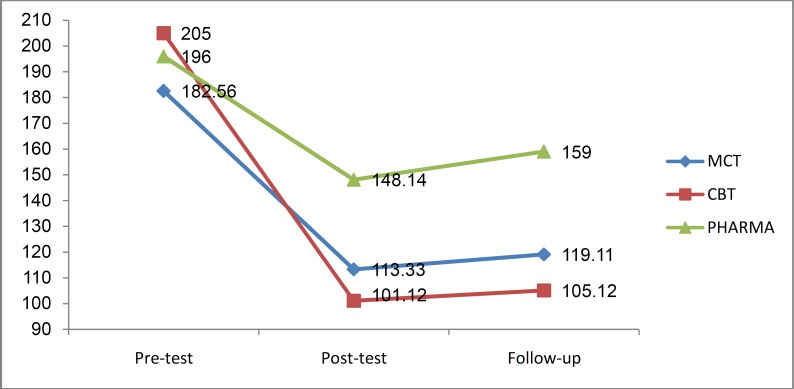
Comparison the adjusted mean Dysfunctional Attitude Scale (DAS) in metacognitive therapy (MCT), cognitive-behavior therapy (CBT), and no psychotherapy groups during three phases of the study

The results of mixed repeated measures ANOVA showed a significant interaction effect between phase and groups (F_ (2, 21, 23.21)_ = 4.08, p = 0.05, η^2 ^= 0.28). According to the figure 3, as well as results of the Post hoc tests for paired comparisons with Bonferroni correction there were no statistically significant differences in dysfunctional thoughts between the groups at pre-test phase. In other words, this indicates the homogeneity of the groups in terms of dysfunctional thoughts. At post-test however, significant differences were observed between the experimental (MCT and CBT) and control groups (p < 0.01). Similar results were noted at the follow-up stage. 

**Figure 4.**
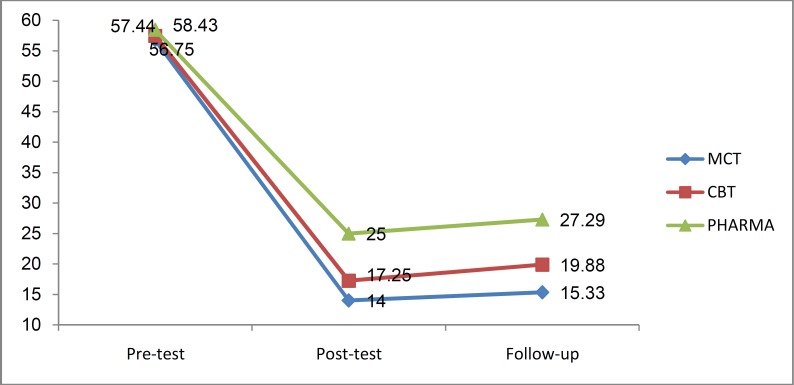
Comparison the adjusted mean Ruminative Response Scale (RRS) in metacognitive therapy (MCT), cognitive-behavior therapy (CBT), and no psychotherapy groups during three phases of the study

Results of mixed repeated measures ANOVA demonstrated a significant interaction effect between phase and groups (F _(4, 42)_ = 73.19, p = 0.05, η^2 ^= 0.43). According to the figure 4, as well as results of the Post hoc tests for paired comparisons with Bonferroni correction there were no statistically significant differences in rumination between the groups at pre-test phase. In other words, this indicates the homogeneity of the groups in terms of rumination. At post-test however, significant differences were observed between the experimental (MCT and CBT) and control groups (p < 0.01). Similar results were noted at the follow-up stage.

## Discussion

This study aimed to investigate and compare the effectiveness of meta-cognitive and cognitive behavioral therapies in treating patients with MDD. 

Results of the study were in accordance with preceding studies (36, 53-55) indicating that meta-cognitive therapy was effective in reducing the severity of depressive symptoms. Wells (24) has suggested that the efficacy of meta-cognitive therapy in reducing the severity of depressive symptoms is related to the recognition of the causes of rumination and the elimination of this maladaptive process. Meta-cognitive approach to depression (24) emphasizes on 1-Positive beliefs about rumination as a means of overcoming depressive feelings and resolving problems; 2-Negative beliefs about the uncontrollability of rumination; 3-Meta-awareness reduction of rumination; and 4-Cognitive attentional syndrome (CAS) (rumination, threat monitoring, maladaptive coping behaviors). Based on this model, depression is maintained and intensified by activation of rumination and maladaptive response patterns (24). Consequently, elimination of ruminations is directly targeted in treatment of depression and practically, positive and negative meta-cognitive beliefs are identified and modified (56). According to the meta-cognitive therapy, the changes in depressed and anxious moods result from the changes in the rumination and related beliefs. Results of the present study showed that the changes observed at post-test were maintained at the follow-up. This could be considered as an evidence for the effectiveness of meta-cognitive therapy in addition with medication compared to mere pharmacotherapy. 

In addition, in line with the findings of preceding studies (57, 58), results of our study showed that the cognitive-behavioral therapy was effective in decreasing the severity of depression. Cognitive-behavioral therapies improve depression through changing and modifying dysfunctional beliefs and cognitive biases. The aim of therapy is to identify and change dysfunctional thoughts and beliefs (59). In CBT, the therapist confronts the negative emotions through reconstruction of client’s thinking process in a way that logical thoughts replace dysfunctional ones (60). Compared to mere pharmacotherapy, the effectiveness of both cognitive behavioral therapy (12, 15) and meta-cognitive therapy (36, 53) has been confirmed in improving symptoms of depression in different studies. However, these two approaches have not been compared with mere pharmacotherapy in depressed patients. Results of this study showed no statistically significant differences between these two approaches in improving symptoms of depression.

Meta**-**cognitive therapy, in line with findings of preceding studies (34, 61) could significantly improve anxiety symptoms of the patients by focusing on basic cognitive features such as rumination, cognitive awareness and meta**-**cognitive thoughts (e.g. worry) which preserve anxiety in patients. According to the meta-cognitive theory, it seems that triggering factor of anxiety is activation of positive meta-cognitive beliefs (for example the worry helps me to cope with problems) and negative meta-cognitive beliefs (such as the worry is not under my control). This model emphasizes on strategies that lead the patients to modify and neutralize these triggering factors in order to control their anxiety. Another factor that plays a role in reducing anxiety symptoms in patients is the reduction of rumination; because rumination affects not only the mood but also leads to cognitive biases, and consequently leads to selective attention of the patients to worrisome issues (54, 62). Rumination causes individuals to have feelings of minimal control over their lives and these feelings are related to increased anxiety (63). The results are in consistent with previous studies (12, 61) showing that cognitive-behavioral therapy is effective on improving symptoms of anxiety through reducing cognitive biases and dysfunctional thoughts. 

In line with a number of previous studies (57), our study demonstrated that CBT was effective in reducing dysfunctional beliefs. The major assumption of this approach is that individuals become vulnerable to depression by experiencing dysfunctional schemas or core negative beliefs about ego and the world (59). Another component of CBT is the systematic biases in thinking style, thinking errors and negative cognitive features. Thus CBT tries to modify and control these thoughts and thinking errors by using cognitive strategies such as identification of automatic negative thoughts and cognitive biases, assessment and questioning the evidences, and exploration contradictory evidences (59). 

As the results showed, MCT can reduce dysfunctional beliefs, even though the magnitude of the reduction was lower than the CBT. Regarding this finding, one can argue that meta-cognitive therapy has improved the dysfunctional thoughts through reducing the rumination and related positive and negative beliefs which are responsible for maintaining dysfunctional thoughts.

The other finding of the study was that the both therapeutic approaches were effective in reducing rumination in depressed patients. The meta**-**cognitive approach conceptualizes the rumination according to a three-level model called self-regulating executive function (S-REF). In this model, rumination is related to self-regulation and emotional dysfunction and is considered a type of coping style with depressed mood. Thus, eliminating rumination is one of the major goals in meta**-**cognitive therapy of depression. This occurs through reduction and alteration in both positive and negative meta**-**cognitive beliefs about rumination and the administration of strategies such as attention control (36). 

One of the main limitations of this study was the fact that the same therapist administrated both meta-cognitive and cognitive behavior therapies, which may have biased the results. It is, therefore, recommended that in the future studies, different therapists conduct the therapeutic interventions. Regarding small sample size of this study, we recommend investigators to conduct similar studies with larger sample size.
